# Early kinetics of CA19-9 and CEA after 5-FU-based chemotherapy for gastrointestinal cancers

**DOI:** 10.1007/s12672-026-05570-4

**Published:** 2026-07-15

**Authors:** Sebastian Lange, Patrick Wenzel, Christof Winter, Jürgen Ruland, Roland M. Schmid, Hana Algül, Michael Quante, Mathias Friedrich

**Affiliations:** 1 TUM School of Medicine and Health, Department of Clinical Medicine-Clinical Department for Internal Medicine II, University Medical Center, Technical University of Munich, 81675 Munich, Germany; 2 Bavarian Cancer Research Center (BZKF), 81675 Munich, Germany; 3 TUM School of Medicine and Health, Institute for Clinical Chemistry and Pathobiochemistry, University Medical Center, Technical University of Munich, 81675 Munich, Germany; 4 TranslaTUM, Center for Translational Cancer Research, Technical University of Munich, 81675 Munich, Germany; 5 German Cancer Consortium (DKTK), Partner Site Munich, a Partnership Between DKFZ and TUM University Hospital, 81675 Munich, Germany; 6 TUM School of Medicine and Health, Department of Clinical Medicine-Comprehensive Cancer Center München, University Medical Center, Technical University of Munich, 81675 Munich, Germany; 7 Clinic for Internal Medicine II, Gastroenterology, Hepatology, Endocrinology, and Infectious Disease, University Hospital Freiburg, 79106 Freiburg, Germany

**Keywords:** Serum tumor markers, CEA, CA19-9, Colorectal cancer, Pancreatic cancer, Biliary tract cancer, Esophageal cancer

## Abstract

**Introduction:**

In gastrointestinal oncology, serum tumor markers such as CEA and CA19-9 are typically monitored over several weeks to assess therapeutic efficacy. The immediate impact of cytotoxic therapy on these serum tumor markers, however, remains poorly characterized.

**Methods:**

We analyzed a single-center cohort of patients with advanced gastrointestinal cancers (pancreatic, biliary, colorectal, and esophageal) treated with 5-fluorouracil-based regimens. Paired serum samples were collected immediately before the start of chemotherapy (0 h) and at the removal of the 48-h 5-FU continuous pump (48 h). The primary endpoint was the percentage change of tumor markers during this time window (∆CEA and ∆CA19-9). Secondary endpoints included the association of these acute kinetics with subsequent radiographic response.

**Results:**

78 cycles of 5-FU-based chemotherapy were included (33 patients, median cycles per patient: 2). CA19-9 increased significantly after chemotherapy (median ∆CA19-9 + 4.8% per patient, Wilcoxon *p* = 0.015; mixed model *p* = 0.062; cycle-level range − 11% to + 92%), as did LDH (median ∆LDH + 12.8% per patient, both Wilcoxon and mixed model *p* < 0.001; cycle-level range − 39% to + 72%). CEA levels remained stable (median ∆CEA − 3.6% per patient, Wilcoxon *p* = 0.09; mixed model *p* = 0.05; cycle-level range − 20% to + 22%). There was no statistically significant association between early changes in serum tumor markers and radiographic outcome.

**Discussion:**

In this hypothesis-generating study, 5-FU-based therapy leads to a statistically significant increase in CA19-9 and LDH levels, but not in CEA, after 48 h. The magnitude of the increase did not predict radiographic response.

**Supplementary Information:**

The online version contains supplementary material available at 10.1007/s12672-026-05570-4.

## Introduction

Gastrointestinal malignancies contribute significantly to cancer-associated mortality. Over the past decades, cytotoxic chemotherapy has been the backbone of managing metastatic disease. Most patients with gastrointestinal cancers are treated with 5-fluorouracil-based combination regimens in either the first- or later-line setting. In metastatic colorectal cancers, this includes 5-FU + Oxaliplatin (FOLFOX) and 5-FU + Irinotecan (FOLFIRI), often combined with agents targeting EGFR or VEGF. In esophageal, junctional, and stomach adenocarcinomas, FOLFOX and FOLFIRI are combined with HER2- or Claudin18.2-directed antibodies or immune checkpoint inhibitors. 5-FU + Oxaliplatin + Irinotecan (FOLFIRINOX) and, more recently, NALIRIFOX (5-FU + Oxaliplatin + liposomal Irinotecan) are frequently used to treat pancreatic ductal adenocarcinoma (PDAC). In the second-line treatment of biliary tract cancers (BTC), FOLFOX can be utilized for biomarker-negative patients.

To guide the care of these patients alongside imaging, serum tumor markers are used to monitor treatment response. In gastrointestinal cancers, carcinoembryonic antigen (CEA), an oncofetal glycoprotein, is elevated in ~ 40% of patients with colorectal cancer [1], ~ 60% of PDAC [2], ~ 50% of stomach and junctional cancers [3], and ~ 40% of BTC [4]. Carbohydrate antigen 19-9 (CA19-9), a sialylated Lewis antigen, is most used for treatment monitoring in metastatic and locally advanced pancreatic and biliary tract cancers (both ~ 80% positive [4, 5]); however, it can be detected in 40% of junctional/stomach [6] and 60% of colorectal cancers [7].

Non-malignant and patient-specific factors can influence both CEA and CA19-9. 5–10% of the population are Le(a-b-) and cannot produce CA19-9 [8]. The liver primarily metabolizes both CEA and CA19-9, so hepatic dysfunction or biliary obstruction can artificially elevate their level. PDAC patients with cholestasis may have elevated CA19-9 levels independent of tumor burden, and successful biliary drainage can cause CA19-9 to fall even without an anti-tumor effect [9].

However, a sustained decline generally correlates with radiographic response and improved survival in metastatic colorectal cancer [10]. An analysis of the PRODIGE 9 trial showed that CEA kinetics predict treatment efficacy, and strong concordance has been reported between CEA ratios and RECIST criteria [11]. Conversely, a continuous rise is almost universally interpreted as a sign of disease progression [12].

A critical exception to this is “flares” or “surges”, transient rises in marker levels shortly after the initiation of chemotherapy. Sørbye and Dahl first described a paradoxical CEA surge in 15% of metastatic CRC patients treated with first-line FOLFOX, noting that these surges peaked at 2 to 8 weeks before declining [13]. Later studies observed that approximately 10% of patients with metastatic CRC, who responded to therapy, experienced a CEA rise of > 20% [14]. A large series from the Royal Marsden found that patients with a transient CEA flare had significantly better response rates and longer survival than those with continuously rising levels [15].

Comparable dynamics have been reported with irinotecan-based therapy and in other malignancies, including gastric cancer and PDAC [16, 17]. The biological mechanism driving these surges is hypothesized to be chemotherapy-induced tumor lysis, in which cell death leads to the release of intracellular antigens into the blood [18].

Despite the recognition of these flares over weeks, the immediate kinetics of tumor markers during the standard 48-h 5-FU infusion window remain uncharacterized. Most studies have assessed levels at intervals of one week or longer. It is currently unknown whether markers begin to rise or fall immediately following a dose of chemotherapy.

Given the limited data available, we conducted an exploratory analysis to characterize the kinetics of CEA and CA19-9 as early as 48 h after chemotherapy. We measured serum CEA, CA19-9, and Lactate Dehydrogenase (LDH) in a cohort of patients at two specific time points: immediately before chemotherapy (0 h) and at the cessation of the continuous 5-FU pump (48 h). We included LDH as a tumor-unspecific complement to CA19-9 and CEA, as it is widely used as a surrogate of cell lysis (e.g., in tumor lysis syndrome and hemolysis), and pre-treatment LDH has independent prognostic value in several gastrointestinal malignancies [19].

Overall, we conducted an exploratory analysis of the kinetics of CEA, CA19-9, and LDH within 48 h of 5-FU-based chemotherapy across a representative spectrum of advanced gastrointestinal cancers. We aimed to determine the proportion of patients exhibiting significant marker changes within this 48-h window and the association between these changes and radiographic outcomes, possibly justifying a larger, prospective, and entity-focused investigation.

## Methods

This observational study included patients treated at the outpatient unit of the Department of Gastroenterology, Hepatology, and Gastrointestinal Oncology at TUM Klinikum rechts der Isar, a tertiary-care university hospital. Inclusion criteria were a histologically confirmed diagnosis of an advanced (metastatic and/or unresectable) gastrointestinal malignancy and undergoing a 5-FU-based chemotherapy regimen between August 2017 and October 2018.

Blood samples were collected at baseline, immediately before the start of chemotherapy administration (0 h), and at removal of the 48-h continuous 5-FU pump (48 h). When monoclonal antibodies were part of the chemotherapy regimen, they were administered after the 5-FU pump was disconnected and blood was drawn for the 48-h time point.

CEA was routinely ordered in all patients with gastrointestinal malignancies. CA19-9 was ordered routinely in pancreatic, biliary, and gastroesophageal cancers but only selectively in colorectal cancers.

Blood samples were processed within 2 h. Serum LDH levels were quantified using a UV assay on a Roche cobas c702 system (Roche Diagnostics, Mannheim, Germany). Serum CEA and CA19-9 levels were quantified using electrochemiluminescence immunoassays on a Roche cobas e801 system (Roche Diagnostics, Mannheim, Germany). Assays followed standardized laboratory protocols with intra- and inter-assay variability ≤ 5%. Assay lower limits of detection were 0.5 ng/ml (CEA), 2 U/ml (CA19-9), and 10 U/l (LDH).

Laboratory and radiologic data were collected from the institutional electronic health record. For radiographic responses, no prospective RECIST 1.1 measurements were available. Therefore, full-length radiology reports from restaging CT were independently reviewed by two clinicians, both with > 10 years of experience in gastrointestinal oncology, who were blinded to tumor marker results and to clinical outcome. Each restaging was classified as regressive, stable, or progressive disease. A scan was classified as progressive when the report described new or enlarging lesions, and as stable when neither clear regression nor clear progression was documented. Discordant classifications were resolved by consensus. Agreement before consensus was 27/29 (93%).

The primary endpoint was the percentage change in each serum marker from pre-infusion (0 h) to post-chemotherapy (48 h), referred to as ∆CEA, ∆CA19-9, or ∆LDH. Because individual patients could contribute multiple chemotherapy cycles, patient-level averages were computed and tested against zero using the one-sample Wilcoxon signed-rank test. Two approaches were used as sensitivity analyses: First, only the first observed cycle per patient was included and tested against zero using the one-sample Wilcoxon signed-rank test. Given the exploratory nature of the study, no formal correction for multiple testing was pre-specified. Benjamini-Hochberg-adjusted q-values are reported alongside raw p-values for the three primary tests. Second, linear mixed-effects models with a random patient intercept were fitted (REML estimation). Intraclass correlation coefficients were calculated using one-way random-effects ANOVA. Model assumptions for the linear mixed-effects models were assessed using QQ plots and residual-versus-fitted plots. Because the distributions of percentage changes were right-skewed, the primary patient-level comparisons used the nonparametric one-sample Wilcoxon signed-rank test. Percentage changes were analyzed on their original scale without transformation.

The relationship between baseline marker levels and the magnitude of the acute percentage change was assessed using Spearman’s rank correlation coefficient.

Comparisons between regimens were evaluated at the cycle level using Fisher’s exact test.

Associations between serum marker changes and favorable (regressive or stable disease) vs. unfavorable (progressive disease) radiographic response were evaluated using the Mann-Whitney U test, restricted to the first cycle per treatment.

For all analyses, a two-sided p-value of < 0.05 was considered statistically significant. Analyses were performed using R (version 4.5.2; R Foundation for Statistical Computing). Descriptive statistics and plots were generated in Prism (version 10.4.1; GraphPad Software).

## Results

### Patient cohort and clinical characteristics

33 patients with a histologically confirmed diagnosis of an advanced (metastatic and/or unresectable) gastrointestinal malignancy, who underwent a 5-FU-based chemotherapy, were included in this study. Paired serum samples were drawn immediately before the initiation of chemotherapy (0 h) and at the cessation of the continuous 5-FU pump (48 h) to study the short-term changes in CEA and CA19-9.

The cohort reflected a typical GI oncology population at a tertiary care center (Table 1 and Supplementary Table 1). 51% of patients were female (17/33). The median age at initial diagnosis was 64 years (range, 38 to 77 years). Cancers included were pancreatic ductal adenocarcinoma (*n* = 11), colorectal adenocarcinoma (*n* = 10), esophageal (*n* = 6), biliary (*n* = 3), duodenal adenocarcinoma (*n* = 1), MiNEN of the colon (*n* = 1), and pancreatic acinar cell carcinoma (*n* = 1). Almost all patients were treated for metastatic disease (31/33; 2 with locally advanced disease), which was either metastatic at initial diagnosis or had relapsed after curatively intended surgery (24 and 7 patients, respectively).

**Table 1:** Baseline demographic and clinical characteristics

Clinical data	No.	%
Patients	33	100
Sex		
Female	17	51.5
Male	16	48.5
Age in years		
Median (range)	64 (38–77)	
Diagnosis		
Pancreatic ductal adenocarcinoma	11	33.3
Colorectal adenocarcinoma	10	30.3
Esophagogastric adenocarcinoma	4	12.1
Biliary tract adenocarcinoma	3	9.1
Esophageal squamous cell carcinoma	2	6.1
Duodenal adenocarcinoma	1	3.0
Pancreatic acinar cell carcinoma	1	3.0
Colon mixed adenoneuroendocrine carcinoma	1	3.0
Intention to treat at studied cycle		
Metastatic at initial diagnosis	24	72.7
Metastatic relapse after surgery	7	21.2
Locally advanced/unresectable	2	6.1
Status of follow-up		
Death	14	42.4
Lost to follow-up	19	57.6
Overall survival in months (death or lost to follow-up)		
Median, (range)	21 (1–121)	

Data are presented as absolute numbers and percentages for categorical variables. Continuous variables are reported as median and range

Overall, 78 cycles of chemotherapy were analyzed (Table 2 and Supplementary Table 2). The median cycles per patient was 2 (range 1–7). Most patients were treated with either irinotecan, oxaliplatin, or both, in combination with 5-FU (FOLFIRI 39.7%, FOLFOX incl. OFF 25.6%, FOLFIRINOX 23.1%). 29 of these chemotherapy cycles (37.2%) included administration of a monoclonal antibody: cetuximab (*n* = 13), bevacizumab (*n* = 8), trastuzumab (*n* = 5), ramucirumab (*n* = 2), or nivolumab (*n* = 1). All antibodies were administered after the 48-h blood draw. For CEA and LDH, data were available for most cycles (98.7% and 96.1%, respectively), whereas only 46.1% of paired CA19-9 data were available. This reflects the universal ordering of CEA for all patients with gastrointestinal cancers at our institution, whereas CA19-9 is usually ordered only for patients with pancreatic, biliary tract, and gastroesophageal cancers. Paired CA19-9 data are therefore concentrated in selected tumor entities (pancreatic adenocarcinoma: 24/28 cycles; biliary tract cancer: 5/5 cycles; colorectal adenocarcinoma: 0/29 cycles) and represent this selected subgroup rather than the whole cohort.

**Table 2:** Characteristics of studied chemotherapy cycles

Chemotherapy cycles	No.	%
Cycles	78	100
Cycles per patient		
Median (range)	2 (1–7)	
Chemotherapy regimens*		
FOLFIRI	31	39.7
FOLFOX (incl. OFF)	20	25.6
FOLFIRINOX	18	23.1
5-FU + lip. Irinotecan + Oxaliplatin	4	5.1
5-FU only	3	3.8
5-FU + lip. Irinotecan	1	1.3
5-FU + Cisplatin	1	1.3
Outcome of studied lines of chemotherapy		
Regressive disease	28	35.9
Stable disease	15	19.2
Progressive disease	28	35.9
Unknown	7	9.0
Completeness of serum markers (0 h and 48 h)		
CEA	77	98.7
CA19-9	36	46.1
LDH	75	96.1
CEA (ng/ml), median (range)		
0 h	20.4 (1-937)	
48 h	19.4 (1–1079)	
∆CEA	− 2.2% (− 19.6% to + 21.6%)	
CA19-9 (U/ml), median (range)		
0 h	282 (2–71,560)	
48 h	288 (2–65,206)	
∆CA19-9	+ 4.5% (− 11.1% to + 92.3%)	
LDH (U/l), median (range)		
0 h	252 (142–546)	
48 h	271 (145–569)	
∆LDH	+ 8.6% (− 39.0% to + 71.9%)	

Data are presented as absolute numbers and percentages for categorical variables. Continuous variables are reported as median and range. Serum marker levels for CEA, CA19-9, and LDH are reported at the pre-infusion (0 h) and post-chemotherapy (48 h) timepoints, ∆ means the calculated change between 0 h and 48 h

*5-FU* 5-Fluorouracil, *CA19-9* carbohydrate antigen 19-9, *CEA* carcinoembryonic antigen, *FOLFIRI* 5-Fluorouracil + Irinotecan, *FOLFIRINOX* 5-Fluorouracil + Irinotecan + Oxaliplatin, *FOLFOX* 5-Fluorouracil + Oxaliplatin, *LDH* lactate dehydrogenase, *OFF* Oxaliplatin + 5-Fluorouracil

*Including 29 cycles (37.2%) with either cetuximab, bevacizumab, trastuzumab, ramucirumab, or nivolumab. All antibodies were administered after the 48-h blood draw

### Early changes in serum markers

**Fig. 1:**
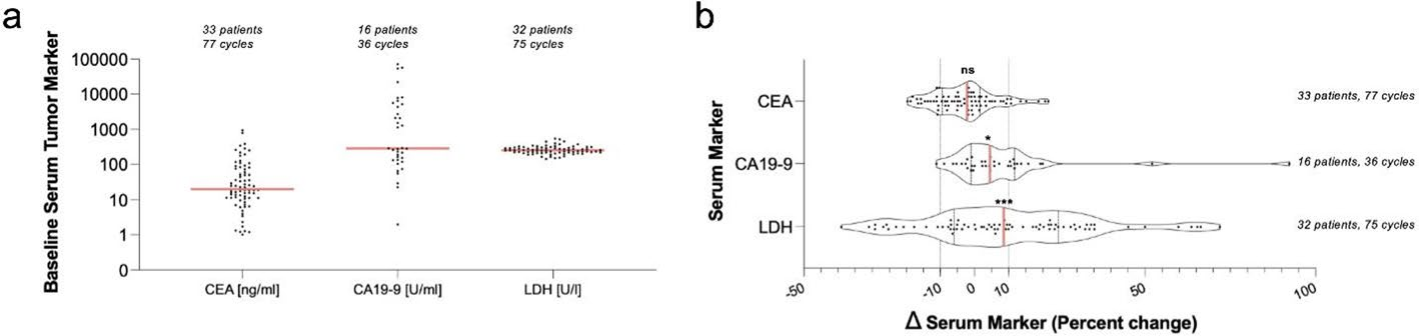
Early kinetics of CEA, CA19-9, and LDH after 5-FU-based chemotherapy. **a** Baseline serum marker levels at 0 h (immediately before chemotherapy) on a log10-scaled y-axis; red dotted line, median. **b** Cycle-level percent change values of ∆CEA, ∆CA19-9, and ∆LDH. Patient-level Wilcoxon tests are indicated: CEA ns (*p* = 0.09), CA19-9 * (*p* = 0.015), LDH *** (*p* < 0.001). Percent change was calculated between time point 48 h (when the continuous 5-FU pump was disconnected) and 0 h (immediately before chemotherapy). *CA19-9* carbohydrate antigen 19-9, *CEA* carcinoembryonic antigen, *LDH* lactate dehydrogenase

There was a significant heterogeneity in baseline tumor marker levels (Fig. 1a; Table 2, and Supplementary Table 2). The median baseline CEA was 20.4 ng/ml (interquartile range: 11.4–69.3, range 1–937) and the median CA19-9 was 282 U/ml (interquartile range: 132–4531, range 2–71,560). To evaluate additional markers of cell death, LDH was included (median 252 U/l, interquartile range 218–299, range 142–546).

After approximately 48 h, the continuous 5-FU pump was disconnected, and serum markers were drawn and measured (Fig. 1b; Table 2, and Supplementary Table 2).

At the cycle level (*n* = 77), the median change in CEA (∆CEA) was small (− 2.2%, interquartile range − 9% to + 1%, range − 20% to + 22%). 23% of paired measurements decreased by < − 10%, and only 9% increased by > + 10%. When averaged per patient (*n* = 33), the median ∆CEA was − 3.6% (interquartile range − 7% to + 1%), which did not differ significantly from zero (Wilcoxon signed-rank test, *p* = 0.09).

In CA19-9, the observed changes were larger. At the cycle level (*n* = 36), the median change (∆CA19-9) was + 4.5% (interquartile range − 1% to + 11%, range − 11% to + 92%). Notably, 12 of 36 (33%) paired measurements showed a CA19-9 increase of > 10%, while only 1 cycle (3%) showed a decrease of > 10%. When averaged per patient (*n* = 16), the median ∆CA19-9 was + 4.8% (interquartile range + 1% to + 12%) and differed significantly from zero (Wilcoxon signed-rank test, *p* = 0.015). This was confirmed when restricting the analysis to the first available cycle per patient only (median + 8.8%, *p* = 0.008). The intraclass correlation coefficient for ∆CA19-9 was 0.48, indicating that nearly half the variance in the acute CA19-9 response is attributable to stable patient-level characteristics.

Changes in LDH (∆LDH) were very similar to CA19-9 changes (Fig. 1b; Table 2). The median change was + 8.6% (interquartile range: − 6% to + 24%, range: − 39% to + 72%) at the cycle-level (*n* = 75). Again, the likelihood of an increase > 10% in the studied cycles was greater than the likelihood of a corresponding decrease (44% vs. 16%). There was CA19-9 data available for 17 cycles in which LDH increased by > 10%; here, CA19-9 levels rose in 76% of samples (in 35% by > 10%), correspondingly, while CEA levels rose in only 35% (in 6% by > 10%). When averaged per patient (*n* = 32), the median ∆LDH was + 12.8% (IQR + 2% to + 24%) and was significant (Wilcoxon signed-rank test, *p* < 0.001). This remained significant in the first cycle-only analysis (*n* = 31, median + 6.85%, *p* = 0.03). In contrast to CA19-9, the ICC for ∆LDH was low (0.05), suggesting that cycle-to-cycle variation in the LDH response is largely not patient-driven.

After Benjamini-Hochberg adjustment of the three primary patient-level Wilcoxon tests, ∆LDH (q < 0.001) and ∆CA19-9 (q = 0.022) remained statistically significant, and ∆CEA remained non-significant (q = 0.09).

In sensitivity analyses using linear mixed-effects models, ∆LDH remained significant (estimate + 11.2%, *p* < 0.001) while ∆CA19-9 and ∆CEA were both borderline (estimate + 7.7%, *p* = 0.062 and estimate − 2.5%, *p* = 0.05, respectively).

The relationship between baseline marker levels and the magnitude of change was assessed using Spearman’s correlation at the patient level. There was a non-significant trend toward an inverse correlation between baseline CA19-9 and ∆CA19-9 (*r*_*s*_ = − 0.36, *p* = 0.18), while no correlation was observed for LDH (*r*_*s*_ = − 0.09, *p* = 0.62) or CEA (*r*_*s*_ = 0.01, *p* = 0.96).

### Exploratory subgroup analysis by tumor entity and chemotherapy regimen

We performed an exploratory subgroup analysis looking at whether changes in tumor markers were confined to specific tumor entities or chemotherapies (Supplementary Table 3). Regarding tumor entity, we focused on pancreatic ductal adenocarcinoma and colorectal adenocarcinoma, which accounted for the majority (73%) of analyzed cycles. In pancreatic cancer (*n* = 28 cycles in 11 patients), cycle-level changes mirrored those of the overall cohort, with median ∆CEA of − 1.8%, median ∆CA19-9 of + 5.8%, and median ∆LDH of + 9.5%. In colorectal cancer (*n* = 29 cycles in 10 patients), the median ∆CEA was − 6.9% and the median ∆LDH was + 8.1%.

Looking at changes stratified by chemotherapy regimen, the cycle-level median ∆CA19-9 was largest with FOLFIRINOX (+ 8.8%, *n* = 18 cycles) compared with FOLFIRI (− 0.7%, *n* = 11). Note that all FOLFIRINOX cycles in this cohort were administered for pancreatic cancer, so this signal cannot be attributed to a specific regimen independently of tumor entity. The median ∆LDH was positive in every oxaliplatin-containing regimen (FOLFIRINOX + 17.1%, FOLFOX + 24.2%, OFF + 37.0%) but near zero under FOLFIRI (− 1.5%). Because 16 of the 18 FOLFOX cycles were administered for non-pancreatic cancers (colorectal, rectal, and esophagogastric), the LDH pattern is not confined to the pancreatic subgroup.

### Individual trajectories

**Fig. 2:**
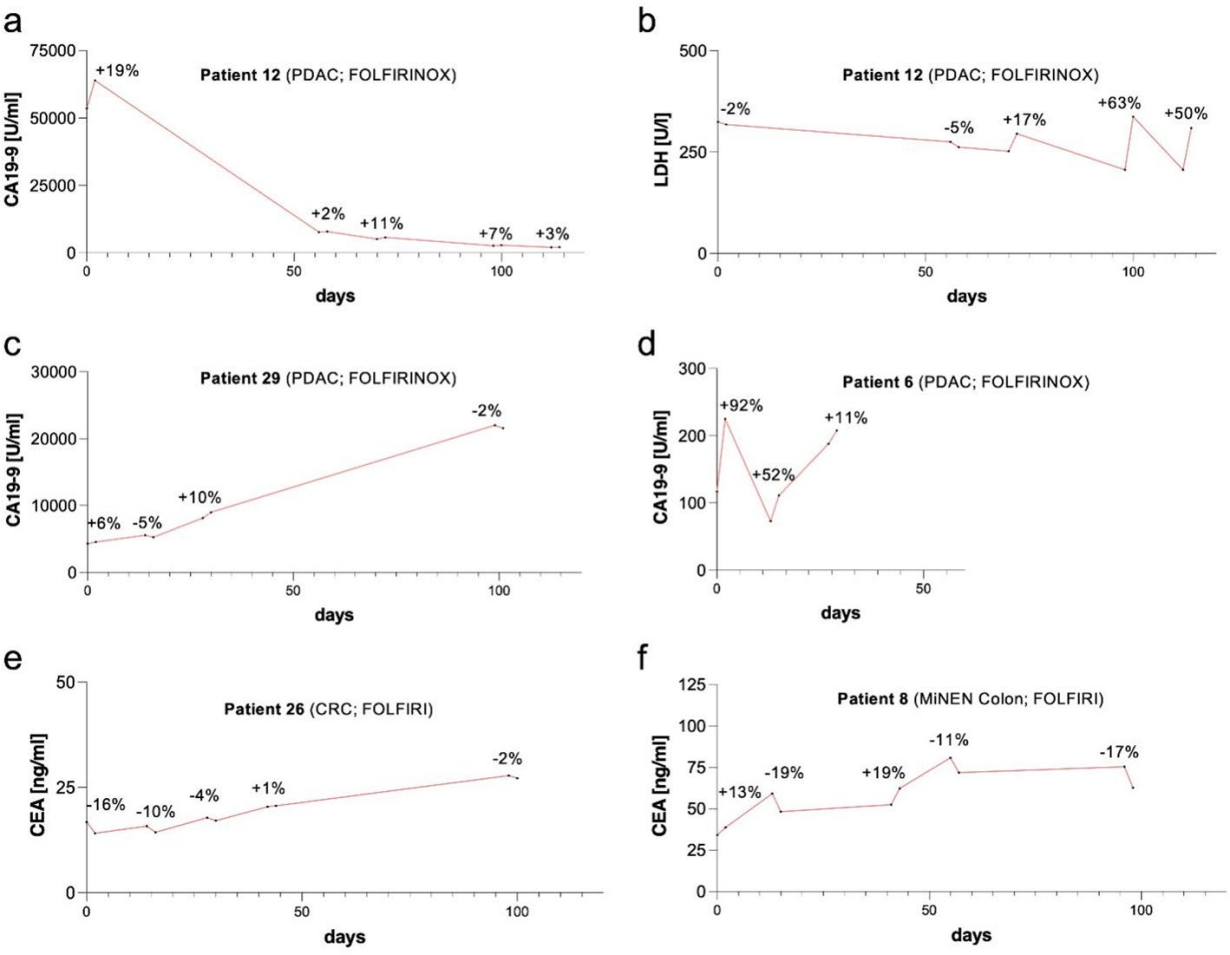
Individual trajectories of CEA, CA19-9, and LDH. **a**–**f** Serum marker courses over the observed treatment interval in six representative patients receiving 5-FU-based chemotherapy for gastrointestinal cancers. **a** and **b** depict the same patient receiving FOLFIRINOX for CA19-9 (**a**) and LDH (**b**). *5-FU* 5-Fluorouracil, *CA19-9* carbohydrate antigen 19-9, *CEA* carcinoembryonic antigen, *CRC* colorectal cancer, *FOLFIRI* 5-Fluorouracil + Irinotecan, *FOLFIRINOX* 5-Fluorouracil + Irinotecan + Oxaliplatin, *LDH* lactate dehydrogenase, *MiNEN* mixed neuroendocrine-nonneuroendocrine neoplasm, *OFF* Oxaliplatin + 5-Fluorouracil, *PDAC* pancreatic ductal adenocarcinoma

As expected, in addition to substantial interpatient heterogeneity, there was substantial intrapatient heterogeneity across several cycles, most likely reflecting tumor response or progression to therapy. For example, in one patient with pancreatic cancer undergoing FOLFIRINOX treatment, CA19-9 decreased from 53,559 to 2090 U/ml over four months. Here, although baseline CA19-9 decreased in each subsequent cycle, there was a positive ∆CA19-9 in each of the five cycles with available data (between + 2% and + 19%) (Fig. 2a). In this patient, there was significant heterogeneity in ∆LDH (− 2% to + 63%) (Fig. 2b).

In another patient with pancreatic cancer undergoing FOLFIRINOX, CA19-9 increased from 4301 to 22,035 over three months (Fig. 2c). In 2/4 analyzed cycles, ∆CA19-9 was positive (+ 6%, + 10%). For patient 6 (Fig. 2d), ∆CA19-9 across all three analyzable cycles was again positive (+ 11% to + 92%), although the absolute change over 40 days was small (117 to 188 U/ml).

There was less variation in ∆CEA overall, with a tendency to negative ∆CEA values (Fig. 1b). In patient 26, who was undergoing FOLFIRI for metastatic colorectal adenocarcinoma, 5/6 analyzed cycles resulted in ∆CEA being negative (− 16% to − 2%), with an overall small increase in CEA (16.8 to 27.2 ng/ml) over the course of three months (Fig. 2e). An outlier example with significant CEA changes is shown in Fig. 2f for a patient with a mixed neuroendocrine-nonneuroendocrine neoplasm (MiNEN) of the colon receiving FOLFIRI.

### Association with radiographic response

We subsequently explored whether the magnitude of these acute marker changes predicted therapeutic efficacy. Radiographic response was assessed on the restaging CT at the end of the planned chemotherapy regimen (usually 8 to 12 weeks). Because formal RECIST evaluations were not available, the full-length radiology reports were classified by two experienced clinicians, and all response-related analyses are exploratory. For patients with multiple time points available during the same line of therapy, we included only the first time point, resulting in 29 of 33 patients (88%) with evaluable radiographic response (Supplementary Table 4).

First-cycle changes were compared between patients with favorable (regressive or stable disease, *n* = 20) and unfavorable (progressive disease, *n* = 9) radiographic response using the Mann-Whitney U test. No significant differences were observed for ∆CEA (median − 0.7% vs. − 0.6%, *n* = 20 vs. 9; *p* = 0.80) or ∆LDH (median + 10.0% vs. + 1.6%, *n* = 19 vs. 8; *p* = 0.45). ∆CA19-9 could not be meaningfully compared because only 3 patients with an unfavorable response (vs. 11 with a favorable response) had evaluable CA19-9 data.

## Discussion

In this single-center study, we investigated the short-term kinetics of serum tumor markers in patients with advanced gastrointestinal malignancies receiving 5-FU-based chemotherapy. Our primary finding is that while CEA levels remain largely stable during the standard 48-h window of the continuous 5-FU pump, CA19-9 and LDH increase significantly.

These transient increases, which occurred independent of the subsequent radiographic response, suggest that they represent a generalized physiologic reaction to cytotoxic stress rather than a specific indicator of tumor sensitivity or resistance.

Because 5-FU is almost always given as part of a multi-agent regimen (FOLFOX, FOLFIRI, FOLFIRINOX, etc.), the observed kinetics should be attributed to the regimen rather than to 5-FU alone. Nearly all patients also received irinotecan and/or oxaliplatin, and some received monoclonal antibodies (however, after the 48-h draw), all of which may influence marker levels. The numerically larger CA19-9 increase under FOLFIRINOX is consistent with, but does not prove, a dose-intensity-related mechanism.

The immediate impact of chemotherapy on serum tumor markers has been a subject of long-standing debate, but limited specific data. Previous literature has described “tumor marker flares” occurring over weeks or months, particularly with hormonal therapies in breast cancer, taxanes in prostate cancer, or platinum-based treatment in colorectal cancer.

However, data regarding the first 48 h of 5-FU infusion are scarce. Our results are consistent with, but do not prove, the hypothesis of chemotherapy-induced tumor lysis, in which rapid cell death releases intracellular antigens into the circulation. The parallel rise in LDH is compatible with this mechanism, but LDH is highly nonspecific and may also increase because of systemic inflammation, acute hepatic or endothelial stress, chemotherapy-related tissue injury, or sample hemolysis. Alternative explanations for the transient CA19-9 increase also cannot be excluded: non-lytic release of intracellular glycoproteins, altered clearance kinetics, and biliary factors, including cholestasis-related CA19-9 elevation independent of tumor burden, could each contribute. Our data cannot distinguish tumor lysis from these alternative causes.

The discrepancy between CA19-9 (which increased) and CEA (which did not) is noteworthy. It may reflect differences in the biological half-life, cellular localization (biliary/ductal vs. membrane-bound), or mechanisms of release between these glycoproteins.

The within-patient consistency of these transient increases varied by marker. The intraclass correlation coefficient for ∆CA19-9 was 0.48, indicating that nearly half the variance in the acute CA19-9 response is attributable to stable patient-level characteristics (e.g., tumor type, chemotherapy regimen, tumor burden). In contrast, the ICC for ∆LDH was only 0.05, suggesting that the LDH response is more stochastic and cycle dependent.

A central message for clinicians is that transient early increases in CA19-9 and LDH within 48 h of chemotherapy may not indicate treatment failure or progression. Although the median increases were modest (+ 4.8% ∆CA19-9 and + 12.8% ∆LDH), individual cycles reached + 92% for ∆CA19-9 and + 72% for ∆LDH within 48 h. If a marker is drawn off-cycle, for example, at pump removal, an isolated rise of this magnitude could be misinterpreted as progression.

It is important to emphasize the hypothesis-generating nature of our findings regarding radiographic response prediction. We found no statistical association between these acute increases and radiographic progression after 8 to 12 weeks. For ∆CEA and ∆LDH, the analyses were reasonably powered (*n* = 29 and *n* = 27, respectively) but non-significant. The ∆CA19-9 analysis was substantially underpowered (*n* = 14, of whom only 3 had an unfavorable response), and clinically meaningful effect sizes cannot be excluded. Currently, clinical decisions, such as switching chemotherapy regimens, should not be based on marker changes observed within this immediate post-infusion window.

Our study has several limitations. First, the analyzed cohort was small (33 patients, 78 cycles) and heterogeneous, encompassing patients with pancreatic (36%), colorectal (33%), esophagogastric (18%), biliary (9%), and duodenal (3%) cancers, which differ in tumor biology and in how tumor markers are used. An exploratory subgroup analysis by tumor type suggests that the CA19-9 signal is primarily driven by the pancreatic subgroup, where CA19-9 is also clinically used. In contrast, the most negative ∆CEA is observed in colorectal cancer. The number of patients with evaluable CA19-9 data was further limited by tumor-type-specific ordering practices at our institution, resulting in the CA19-9 analyses being based on a selected subgroup enriched for pancreatic and biliary cancers, and generalized conclusions regarding CA19-9 across all gastrointestinal cancers cannot be drawn from these data.

Second, the timing of sample collection at 48 h was dictated by the logistical workflow of pump removal. A significant proportion of our patient population opted for pump removal by their general practitioner or self-removal at home, potentially leading to selection bias, as only patients returning to the clinic (e.g., for toxicity management or monoclonal antibody administration) were included.

Third, radiographic response was classified from full-length reports rather than prospective RECIST 1.1 measurements. Although an independent review with blinding to marker values and clinical outcome was applied, this approach introduces subjectivity, and the response-related findings should be regarded as exploratory.

Finally, while we may hypothesize that tumor lysis is the mechanism, we lacked more direct biological correlates (e.g., cytokines, uric acid, apoptosis markers, or circulating tumor DNA) to distinguish cell death from non-specific release. Future research should compare these transient increases against circulating tumor DNA dynamics to determine if liquid biopsy can better distinguish between benign “marker flares” and true disease progression, potentially superseding traditional markers in this acute setting.

This study provides the first detailed characterization of the very early kinetics of serum tumor markers during 5-FU therapy. Given the retrospective nature and sample size, our results should be considered hypothesis-generating and warrant confirmation in larger, prospective cohorts.

Nevertheless, we identified a frequent, transient increase in CA19-9 and LDH that does not appear to correlate with treatment failure. Awareness of this phenomenon is important to prevent premature discontinuation of potentially effective therapies due to rising tumor marker levels.

## Supplementary Information

Below is the link to the electronic supplementary material.

Supplementary Material 1. Supplementary Table 1: Detailed baseline demographic and clinical characteristics. Supplementary Table 2: Individual chemotherapy cycles. Supplementary Table 3: Cycle-level changes by tumor entity and chemotherapy. Supplementary Table 4: Patient-level analysis and radiographic response.

## Data Availability

The datasets supporting the conclusions of this article are included within the article and the supplementary tables.
